# A Comparison of Ku0063794, a Dual mTORC1 and mTORC2 Inhibitor, and Temsirolimus in Preclinical Renal Cell Carcinoma Models

**DOI:** 10.1371/journal.pone.0054918

**Published:** 2013-01-22

**Authors:** Hao Zhang, Dror Berel, Yanping Wang, Ping Li, Neil A. Bhowmick, Robert A. Figlin, Hyung L. Kim

**Affiliations:** 1 Department of Surgery/Division of Urology, Cedars-Sinai Medical Center, Los Angeles, California, United States of America; 2 Biostatistics Core, Cedars-Sinai Medical Center, Los Angeles, California, United States of America; 3 Department of Medicine/Division of Hematology and Oncology, Cedars-Sinai Medical Center Los Angeles, California, United States of America; 4 Samuel Oschin Comprehensive Cancer Institute, Cedars-Sinai Medical Center, Los Angeles, California, United States of America; Children’s Hospital Boston & Harvard Medical School, United States of America

## Abstract

Rapamycin analogs, temsirolimus and everolimus, are approved for the treatment of advance renal cell carcinoma (RCC). Currently approved agents inhibit mechanistic target of rapamycin (mTOR) complex 1 (mTORC1). However, the mTOR kinase exists in two distinct multiprotein complexes, mTORC1 and mTORC2, and both complexes may be critical regulators of cell metabolism, growth and proliferation. Furthermore, it has been proposed that drug resistance develops due to compensatory activation of mTORC2 signaling during treatment with temsirolimus or everolimus. We evaluated Ku0063794, which is a small molecule that inhibits both mTOR complexes. Ku0063794 was compared to temsirolimus in preclinical models for renal cell carcinoma. Ku0063794 was effective in inhibiting the phosphorylation of signaling proteins downstream of both mTORC1 and mTORC2, including p70 S6K, 4E-BP1 and Akt. Ku0063794 was more effective than temsirolimus in decreasing the viability and growth of RCC cell lines, Caki-1 and 786-O, *in vitro* by inducing cell cycle arrest and autophagy, but not apoptosis. However, in a xenograft model there was no difference in the inhibition of tumor growth by Ku0063794 or temsirolimus. A potential explanation is that temsirolimus has additional effects on the tumor microenvironment. Consistent with this possibility, temsirolimus, but not Ku0063794, decreased tumor angiogenesis *in vivo,* and decreased the viability of HUVEC (Human Umbilical Vein Endothelial Cells) cells *in vitro* at pharmacologically relevant concentrations. Furthermore, expression levels of VEGF and PDGF were lower in Caki-1 and 786-O cells treated with temsirolimus than cells treated with Ku0063794.

## Introduction

Renal cell carcinoma (RCC) is the most common malignancy of the kidney. It’s the seventh most common cancer in males and the ninth most common cancer in females, with a worldwide incidence of over 210,000 cases, resulting in 102,000 deaths per year [1]. RCC is refractory to traditional cytotoxic chemotherapy and radiotherapy [2]. Recently, treatment options for advanced RCC have been expanded by the approval of molecularly-targeted inhibitors of protein kinases. An important molecular target for RCC is the mechanistic target of rapamycin (mTOR), which is a pivotal regulator of cell proliferation and survival [3].

The mTOR protein is a serine/threonine kinase that forms two functionally unique complexes: mTOR complex 1(mTORC1) and mTOR complex 2 (mTORC2). mTORC1 function is mediated through phosphorylation of S6K1 and 4E-BP1, which stimulate mRNA translation and growth [4]. When energy is abundant, mTORC1 actively suppresses autophagy. Autophagy is a survival mechanism that allows cells to survive nutrient deprivation by using self-components as a source of energy [5]. mTORC2 was first identified as a regulator of actin cytoskeleton. More recently, mTORC2 has been shown to phosphorylate members of the AGC kinase families, including Akt. Increased Akt activity has been linked to various diseases, including cancer and diabetes [6], [7]. Therefore both mTORC1 and mTORC2 are rational targets for anti-cancer treatments.

The U.S. Food and Drug Administration (FDA) has approved two mTOR inhibitors, temsirolimus and everolimus, for the treatment of RCC. The approved mTOR inhibitors produce clinically meaningful responses, however, the responses are short-lived and almost never curative [8]–[11]. Both temsirolimus and everolimus are rapamycin analogs that target mTORC1 but not mTORC2. Therefore, it has been argued that strategies to target mTORC1 and mTORC2 may produce better clinical responses [7]. Furthermore, it has been proposed that drug resistance develops due to compensatory activation of mTORC2 signaling during treatment with temsirolimus or everolimus [12]. This argument is supported by the observation that selective inhibition of mTORC1 can increase Akt activity by removing negative feedback loops provided by mTORC1, S6K1, and IRS1 [4]. Several synthetic small molecules have been described that inhibit both mTORC1 and mTORC2 and some are already in early phase clinical trials [7], [13]–[15]. Ku0063794 is a highly specific small-molecule inhibitor of mTOR kinase that inhibits both mTORC1 and mTORC2 [16]. Ku0063794 inhibits the phosphorylation of S6K1 and 4E-BP1, which are downstream substrates of mTORC1, and it inhibits Akt phosphorylation on Ser473, which is the target of mTORC2.

We evaluated Ku0063794, in parallel with temsirolimus, as potential treatments for RCC using *in vitro* and *in vivo* models. Expression profiles confirmed that genes associated with both mTORC1 and mTORC2 were enriched in clear cell RCC. We confirmed that Ku0063794 inhibits mTORC1 and mTORC2 in RCC. We showed that Ku0063794 suppresses cell viability and growth *in vitro* by inducing cell cycle arrest and autophagy, but not apoptosis. Ku0063794 significantly decreased the growth of RCC tumors in a mouse xenograft model and blocked mTOR activity *in vivo*. However, Ku0063794 was no more effective in inhibiting tumor growth *in vivo* than temsirolimus. A potential explanation for this unexpected finding is that temsirolimus inhibits angiogenesis while Ku0063794 does not, suggesting that an increase in direct antitumor effect is offset by a lack of antiangiogenic effect in the tumor microenvironment.

## Materials and Methods

### mTOR Pathway Analysis

To identify mTOR pathway genes, Majumder et al compared the expression profiles of prostate from AKT1-Tg (transgenic) mice that overexpress human AKT and WT (wildtype) prostate [17]. The mTOR pathway genes were divided into those that were sensitive and insensitive to a rapalog, everolimus. Rapalog insensitive genes were presumed to be related to mTORC2 signaling. Creightion used these gene sets to identify 57 genes that positively correlated with Akt mRNA in human breast tumors [18]. To assess the association of these 57 genes with kidney tumors, whole genome expression data for ccRCC (clear cell renal cell carcinoma) was obtained from the NCBI GEO repository (GSE6344) [19]. In an analysis comparing ccRCC and matched normal kidney, differentially expressed genes were selected using ‘significance analysis of microarrays’ (SAM) method [20]. Enrichment analysis of the 57 mTOR pathway genes was performed using the Fisher exact test.

### Cell Culture and Reagents

All cell lines were obtained from American Type Culture Collection. Caki-1 cells were maintained in McCoy’s 5A medium (Invitrogen) and 786-O cells were maintained in RPMI medium 1640 (Invitrogen) at 37°C in 5% CO2. All media were supplemented with 10% fetal bovine serum (FBS), 10 mM HEPES buffer (pH 7.2–7.5, Invitrogen), 100 units/ml penicillin (Invitrogen) and 100 µg/ml streptomycin (Invitrogen). Ku0063794 (Chemdea) and temsirolimus (LC Laboratories) were solubilized in dimethyl sulfoxide (DMSO). All antibodies were purchased from Cell Signaling Technology except the antibody against β-actin, which was purchased from Sigma-Aldrich.

### Cell Lysis and Western Blots

For the intracellular signaling study, the Caki-1 and 786-O cells were grown to 90% confluency and then treated with Ku0063794, temsirolimus or DMSO for various lengths of time ranging from 10 minutes to 3 hours. The cells were washed with ice-cold PBS before being treated with detergent lysis buffer (50 mM Tris-HCl pH 7.5, 150 mM NaCl, 1 mM EDTA, 10 mM DL-Dithiothreitol, 10% glycerol, 0.5% Sodium Deoxycholate, 1% Triton-X100) supplemented with 10 mM NaF, protease inhibitors (1∶100 dilution of protease inhibitor cocktail from SIGMA-Aldrich) and phosphatase inhibitors (1∶100 dilution of phosphatase inhibitor cocktail 3 from SIGMA-Aldrich). The proteins in the cell lysate were precipitated with acetone and then dissolved in 2X SDS sample buffer (100 mM Tris-HCl pH6.8, 2% SDS, 10% glycerol, 5% β-mercaptoethanol, 4 M urea, 2 mM EDTA, and 0.01% bromophenol blue). Protein concentrations were measured with the Bio-Rad Protein Assay to ensure consistent protein-loading onto SDS-PAGE. Nitrocellulose membranes were used for the protein transfer and western blots were performed according to recommendations of the antibody manufacturers. Western blots were quantified with ImageJ software (version 1.47a).

### Cell Viability Assay

The cell viability assay was performed with the CellTiter-Glo® Luminescent Cell Viability Assay Kit (Promega) in 96-well clear-bottom tissue-culture plates (Corning) as recommended by the manufacturer. The Caki-1, 786-O or HUVEC cells were plated at densities low enough to ensure that cells never reach full confluency. A day after plating the cells, drug (Ku0063794 or temsirolimus) or vehicle (DMSO) was added at the indicated concentrations in triplicate wells. Cell viability was measure after 24, 48, 72 and 96 hours of treatment. Luminescence was measured with the Wallac 1420 VICTOR^2^™ plate reader (PerkinElmer). Cell viability is presented as the percentage of the corresponding negative control at each time point. Inhibitory concentrations (e.g. IC_50_, IC_30_ and IC_20_) were calculated using Graphpad Prism (version 6.0).

### Flow Cytometric Analysis of Cell Cycle Distribution

Caki-1 and 786-O cells were plated in 10 cm cell culture dishes to allow the untreated control to reach 50% confluency by the end of the experiment. A day after plating the cells, the drug (Ku0063794 or temsirolimus) or vehicle (DMSO) was added at the indicated concentrations in triplicate wells. After 72 hours of treatment, live cells in each dish were counted. To assess cell-cycle distribution, cells were resuspended in 70% ethanol (v/v). The cells were stained for 1 hour in the dark with PBS containing 50 µg/ml propidium iodide and 50 µg/ml RNase A. The DNA content of the cells was measured with the FACS Calibur flow cytometer (BD Bioscience) and the CellQuest software. The cell-cycle distribution was determined using Modfit LT software.

### Autophagy and Apoptosis Analysis

For the autophagy study, Caki-1 and 786-O cells were pretreated with 10 µg/ml pepstatin A and 10 µg/ml E-64d for 90 minutes, and then treated with Ku0063794 (1 µM or 2 µM) or temsirolimus (300 nM or 1 µM) for 24 hours in the presence of 10 µg/ml pepstatin A and 10 µg/ml E-64d. Cell lysates were loaded onto SDS-PAGE and blotted for LC3. To detect the conversion of LC3-1 to LC3-2, which occurs during autophagy, protease inhibitors (pepstatin A and E-64d) are added to prevent degradation of LC3-2 [21]. For apoptosis analysis, Caki-1 and 786-O cells were treated with Ku0063794 (1 µM or 2 µM) or temsirolimus (300 nM or 1 µM) for 24 hours or 48 hours. At the end of the treatment, the cells were trypsinized, resuspended, and then double stained with propidium iodide and FITC-conjugated Annexin V using the Annexin V apoptosis detection kit (Santa Cruz Biotechnology). Cells were also treated in parallel with 20 mM H_2_O_2_ for 30 minutes as a positive control. Staining was measured with the FACSCalibur flow cytometer (BD Bioscience) and analyzed with the CellQuest software.

### Xenograft Model

Six-week-old female, Nu/Nu nude mice were purchased from Charles River Laboratories. Approximately 5×10^6^ 786-O cells were injected subcutaneously into the flank, and the tumors were allowed to reach 5 mm in diameter before starting treatment. The mice were randomly divided into three groups and treated once daily (five days a week) by intraperitoneal (IP) injection with DMSO (vehicle control), temsirolimus (0.6 mg/kg), or Ku0063794 (8 mg/kg). The tumor size and body weight were measured at least twice weekly. Tumor volume was estimated using the standard formula: (length×width^2^)/2. The mice were sacrificed after 46 days of treatment and the tumors were excised. Tumors were divided and either flash frozen in liquid nitrogen or placed in 10% buffered formalin and paraffin embedded (PE). The flash frozen tumors were homogenized in detergent lysis buffer with tissue homogenizer. The supernatant was used for western blotting. To prepare drugs for injection, temsirolimus was solubilized as a 5 mM stock solution in DMSO. Prior to IP injection, temsirolimus was diluted (15 µg/100 µl) in PEG1500 (50% (w/v) in 75 mM Hepes, pH 8.0, Roche Applied Science). Ku0063794 was solubilized in one part DMSO and then diluted (200 µg/100 µl) with 4 parts PEG1500 (50% (w/v) in 75 mM Hepes, pH 8.0, Roche Applied Science). All animal experiments were conducted with approval of the Institutional Animal Care and Use Committee (IACUC003011).

### Immunohistochemistry

PE (paraffin-embedded) tumors were cut to 4 µm sections, deparaffinized in xylene, and rehydrated in a graded series of ethanol and PBS. For CD34 staining, the slides were incubated with citrate buffer (pH 6.0; Zymed) at 95°C for 30 minutes to expose the antigen. Sections were immersed in peroxidase and alkaline phosphatase blocking reagent (Dako). Sections were then incubated overnight at 4°C with CD34 primary antibody (MEC14.7, Abcam) in antibody diluting buffer (Antibody Diluent, Dako). After washing with TBS-T (Tris-Buffered Saline Tween-20), sections were incubated with secondary antibody for 30 minutes (Anti-Rat Ig ImmPRESS^TM^ Reagent Peroxidase, Vector). After washing with TBS-T, the immune complex was visualized using DAB substrate solution (Liquid DAB + Substrate Chromogen System, Dako). The digital images were captured at 200x magnification using Nikon Optiphot-2 microscope with a Nikon Digital Sight DS-L1 camera system. For each tumor section, 8 random fields (0.36 mm^2^ each) were examined to determine the microvessel density.

### Quantitative RT PCR

Caki-1 and 786-O cells were treated with 2 µM Ku-0063794, 300 nM temsirolimus, or DMSO (control) for 24 hours. Total mRNA was extracted with the MasterPure RNA purification kit (Epicentre) following the manufacturer’s instructions. cDNA was generated with the High Capacity cDNA reverse transcription kit (Invitrogen). TaqMan® PCR was performed as previously described [22]. Briefly, cDNA generated from 1 ng of total RNA was used in each PCR reaction containing TaqMan® universal PCR master mix (Applied Biosystems). Predesigned TaqMan® primer and probe sets based on 5− nuclease chemistry using TaqMan® minor groove binder (MGB) probes were ordered (Applied Biosystems). For some genes, TaqMan® assays were custom-designed. The cycle thresholds (C_T_) were normalized using 3 reference genes: TFRC, B2M and TBP (ΔC_T_ = C_T_ (test gene) − C_T_ (mean for the reference genes)) [23]. See Table S1 for primer/probe sequences and assay ID’s. All expressions were converted to linear values prior to statistical analysis.

### Statistical Analysis

In the xenograft model, tumor sizes in the treatment groups were compared using the Kruskal-Wallis test. Continuous variables were compared using the Wilcoxon rank sum test. P<0.05 was considered significant. The pathway analysis was performed using the R (version 2.13.0)/ Bioconductor (version 2.8) software.

## Results

### mTOR Pathway is Activated in Clinical Renal Tumors

**Figure 1:**
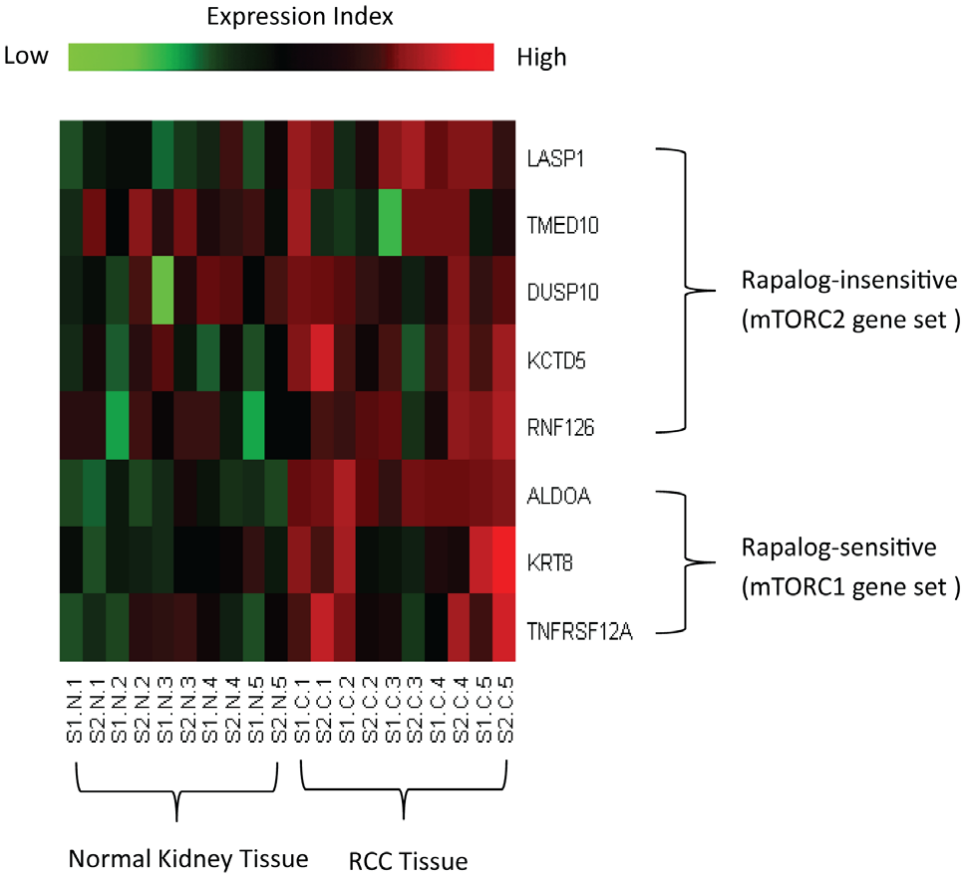
The mTOR pathway genes are overexpressed in ccRCC. A heat map was generated with mTORC1 and mTORC2 genes that were significantly over-expressed in ccRCC when compared to normal kidney. Creighton examined genes induced by Akt using transgenic mice overexpressing AKT and identified 57 mTOR pathway genes that were positively correlated with AKT expression in human breast cancer (Oncogene, 26∶4648-55). The expression of these mTOR pathway genes, reported as rapalog sensitive (mTORC1) or insensitive (mTORC2), were examined in ccRCC.

The mTOR pathway was activate in RCC when expression profiles of tumor and adjacent normal kidney were compared (Fig. 1). A SAM analysis was performed using whole genome expression profiles generated by Tun et al [19]. Genes associated with both the mTORC1 and mTORC2 pathways were enriched (Fisher exact test, p-value = 0.01) in human clear cell RCC, providing a rationale for targeting both pathways with second generation mTOR inhibitors.

### Ku0063794 Inhibits the Activity of mTORC1/2 *in vitro* in RCC Cell Lines

**Figure 2:**
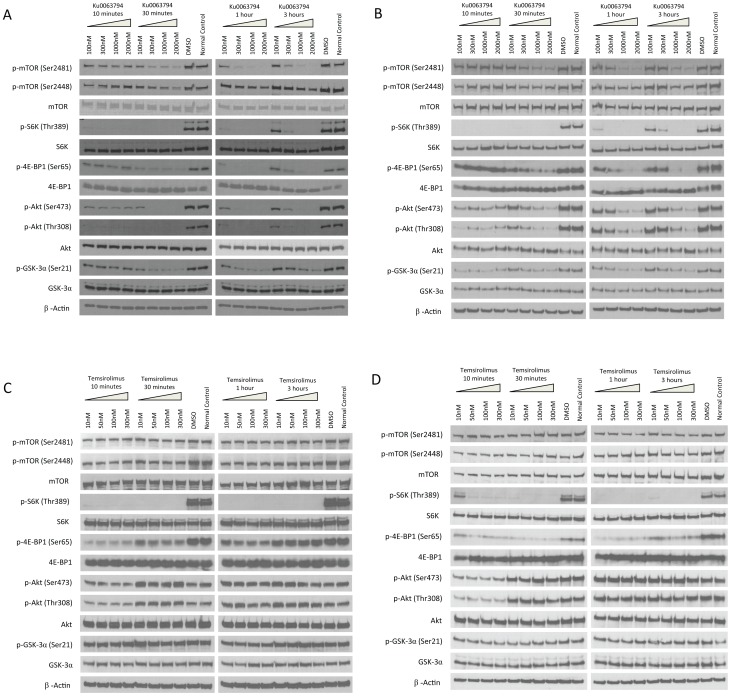
Intracellular signaling in RCC cells treated with Ku0063794 or temsirolimus. Caki-1 (**A**) and 786-O (**B**) cells were treated with Ku0063794 at the indicated concentrations for the indicated length of time. Control cells treated with DMSO were treated for 3 hrs. Cell lysates were used for western blotting to analyze the mTORC1/2 pathway. In an analogous experiment, Caki-1 (**C**) and 786-O (**D**) cells were similarly treated with temsirolimus or DMSO. The figure is representative of triplicate experiments.

Ku0063794 was reported to be a dual inhibitor of mTORC1 and mTORC2 in HEK-293 cells [16]. To investigate whether the same inhibitory effects also exist in human RCC cell lines, Caki-1 and 786-O cells were treated at increasing concentrations of Ku0063794 for various lengths of time (10 minutes to 3 hours) *in vitro*. Ku0063794 was compared to temsirolimus, which is a rapamycin analog that is approved for treating advanced RCC. Cell lysates were used for western blots to analyze the activities of mTORC1/2 and their downstream effectors. Ku0063794 inhibited both mTORC1 and mTORC2 as indicated by the decrease in phosphorylation of downstream effectors. The phosphorylation of Thr389 on p70 S6K and Ser65 on 4E-BP1, which are both phosphorylated by mTORC1, were inhibited by Ku0063794 in both Caki-1 and 786-O cells (Fig. 2A & 2B). mTORC2 kinase activity was also inhibited by Ku0063794; phosphorylation of Thr308 and Ser473 on Akt and Ser21 on GSK-3α (the target phosphorylation site of Akt) were inhibited by Ku0063794 in 786-O and Caki-1 cells (Fig. 2A & 2B). The phosphorylation of mTOR itself on Ser2448 and Ser2481 decreased in both cell lines when treated with Ku0063794.

When Caki-1 and 786-O cells were treated with temsirolimus, the phosphorylation of targets downstream of mTORC1 (Thr389 on p70 S6K and Ser65 on 4E-BP1) decreased (Fig. 2C &2D). However, there was no consistent effect on phosphorylation of targets downstream of mTORC2 such as Ser473 on Akt and Ser21 on GSK-3α (Fig. 2C & 2D), confirming that temsirolimus is an inhibitor for mTORC1, but not mTORC2. The western blot results are summarized in Table S2. The western blots for 1-hour treatment of both cell lines with both drugs were quantified (Fig. S1 & S2).

### Ku0063794 Suppresses the Viability and Proliferation of RCC Cell Lines

**Figure 3:**
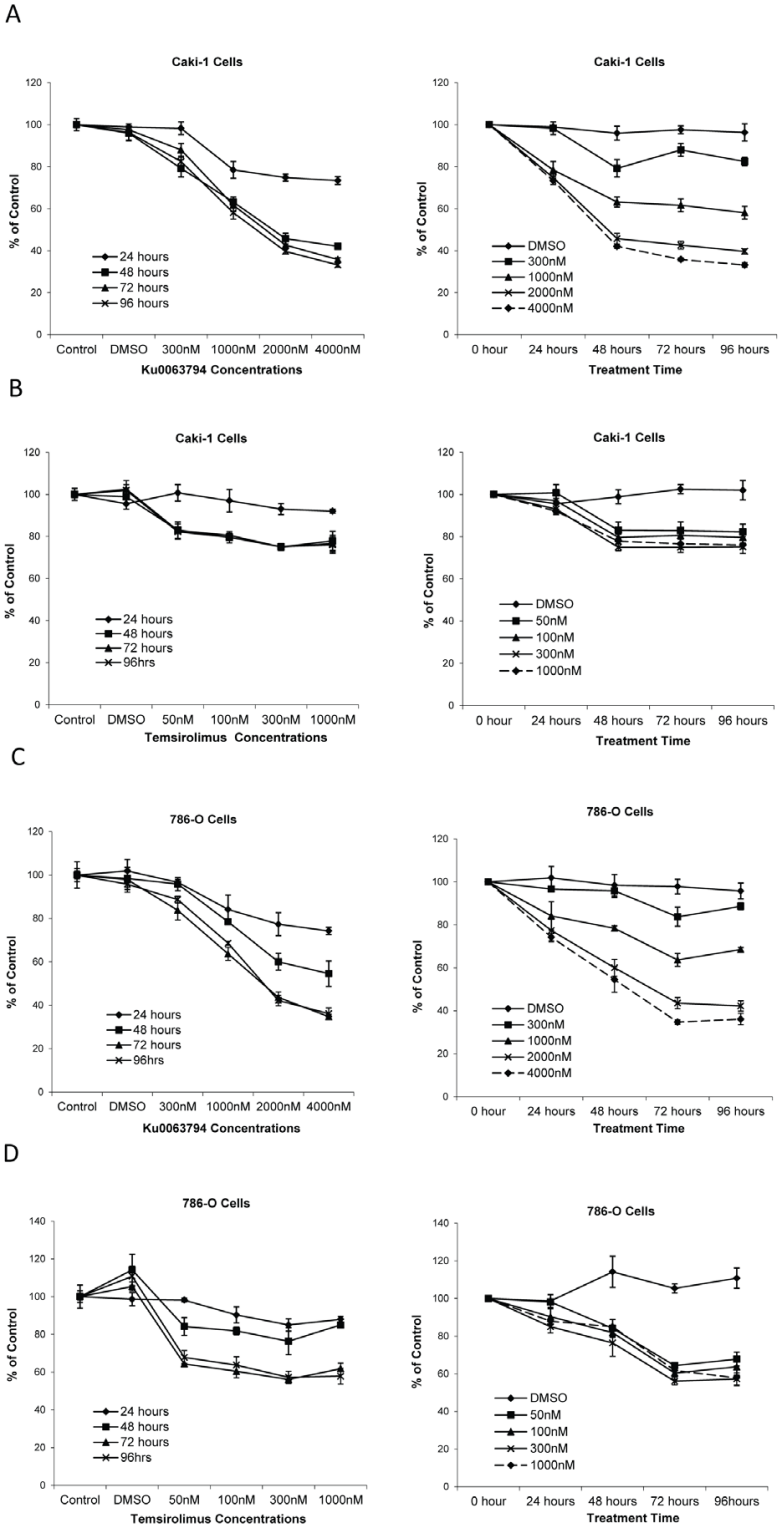
Ku0063794 and temsirolimus decreased the viability of RCC cells. Caki-1 (**A**, **B**) and 786-O (**C**, **D**) cells were plated on 96-well tissue-culture plates and then treated with Ku0063794 (**A**, **C**) or temsirolimus (**B**, **D**) at the indicated concentrations or DMSO (vehicle control) for 24 to 96 hours. Cell viability was calculated as a percent of the control and reported as a function of treatment time and drug concentration. The error bars indicate the standard error of the mean for experiments performed in triplicate.

To assess the effect of Ku0063794 on cell viability, Caki-1 and 786-O cells were treated with Ku0063794 or temsirolimus at increasing concentrations for various lengths of time, from 24 hours up to 96 hours. Cell viability was measured at 24 hours intervals. Both Ku0063794 and temsirolimus decreased the viability of RCC cells (Fig. 3 & Table S3). However, there was a direct correlation between Ku0063794 concentration and cell viability over a greater range of concentrations (Fig. 3A & 3C) when compared to temsirolimus (Fig. 3B & 3D). There was little additional effect on viability of either Caki-1 or 786-0 cells when temsirolimus concentrations were increased from 100 nM to 1 µM.

**Figure 4:**
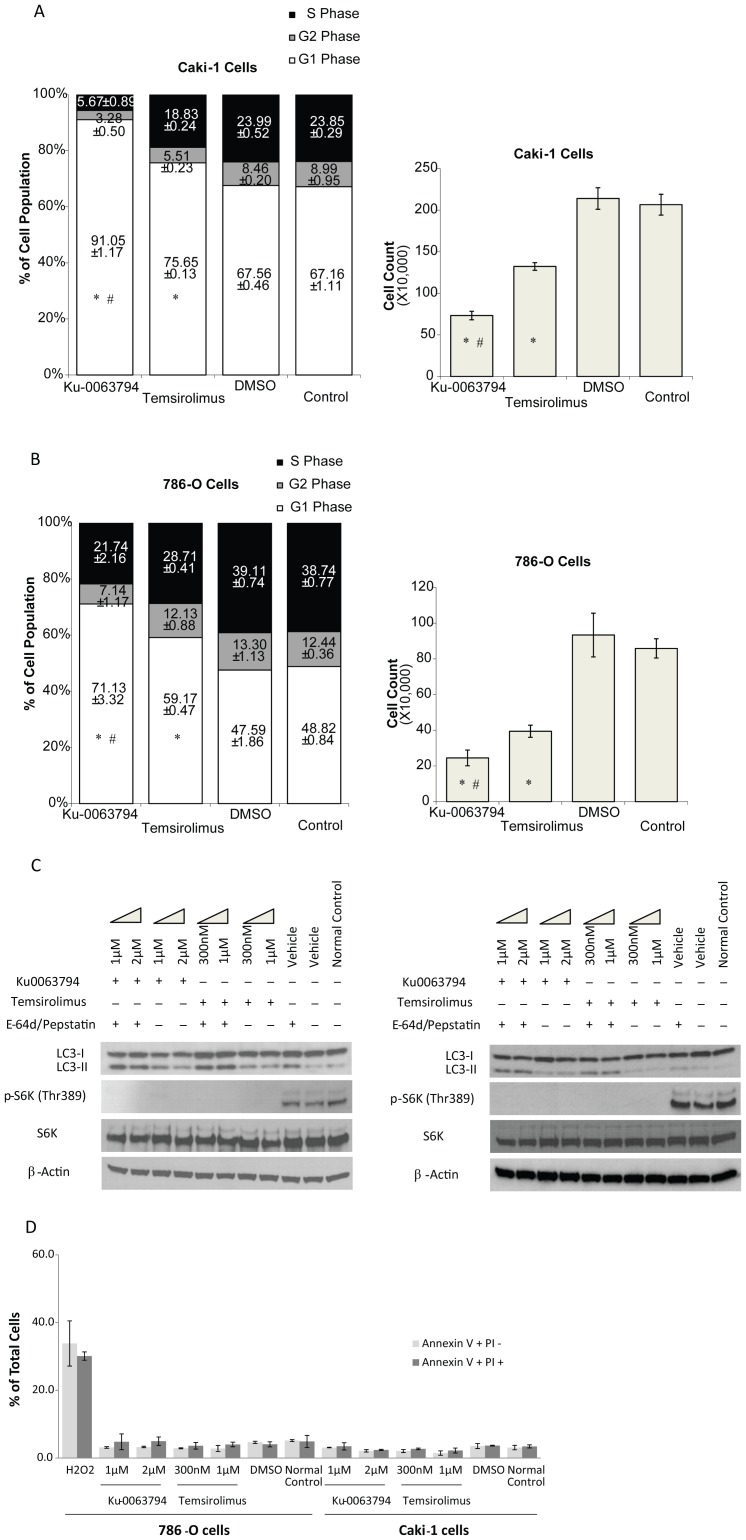
Ku0063794 and temsirolimus induced G1 cell cycle arrest and autophagy in RCC cells. Caki-1 (**A**) and 786-O (**B**) cells were plated and treated with 2 µM Ku0063794, 300 nM temsirolimus or DMSO (vehicle control) for 72 hours. The treated cells were subjected to cell cycle analysis (**left**). Cells were trypsinized and live cells were counted (**right**). The percent of cells at each cell cycle and the standard error of the mean are provided. *p<0.01 comparing either Ku0063794 or temsirolimus treatment to either DMSO treatment or untreated control. #p<0.01 comparing Ku0063794 to temsirolimus. Ku0063794 and temsirolimus induced autophagy in RCC cells as indicated by the increase in LC3-2/LC3-1 ratio. (**C**). Caki-1 (**left**) and 786-O (**right**) cells were treated with Ku0063794 or temsirolimus at the indicated concentration, or DMSO (vehicle control) for 24 hours with/without pepstatin A and E-64d. The figure is representative of triplicate experiments. (**D**) Ku0063794 and temsirolimus failed to induce apoptosis in RCC cells. Annexin-V and propidium iodide staining was performed following Ku0063794 or temsirolimus treatment at the indicated concentrations for 24 hours. As a positive control, 786-O cells were treated with 20 mM H_2_O_2_ for 30 minutes. Error bars indicate the standard error of the mean for triplicate experiments.

Effects of Ku0063794 and temsirolimus on cell cycle distribution were investigated in RCC cell lines. Treatment with either drug led to cell cycle arrest, with greater percentage of cells in G1 phase (Fig. 4A & 4B, left panels). To confirm that cell cycle arrest produced a decrease in cell proliferation, cell counts were assessed in the same experiment (Fig. 4A & 4B, right panels). Cell cycle was assessed after 72 hours of drug-treatment since maximal decrease in cell viability was noted at this time point (Fig. 3). At the concentrations examined, Ku0063794 exhibited stronger induction of G1 phase arrest and greater inhibition of cell growth than temsirolimus.

### Ku0063794 Induces Autophagy but not Apoptosis in RCC Cell Lines

Autophagy and apoptosis were investigated as potential mechanisms leading to cell death. During autophagy, LC3 (microtubule-associated protein 1 light chain 3) is converted by a process of lipidation from LC3-1 to LC3-2, which is a marker for autophagy. LC3-2 is rapidly degraded in all cells, and pepstatin A and E-64d are added to allow measurement of LC3-2 production. We found that when Caki-1 cells were treated with Ku0063794 for 24 hours, the ratio of LC3-2/LC3-1 increased in the presence of pepstatin A and E-64d, and when 786-O cells were treated with either Ku0063794 or temsirolimus for 24 hours, the ratio of LC3-2/LC3-1 increased in the presence of pepstatin A and E-64d (Fig. 4C & Fig. S3 & S4), indicating that Ku0063794 may be more effective than temsirolimus in inducing autophagy. Apoptosis is another mechanism that leads to cell death. Caki-1 cells or 786-O cells were double stained with FITC-Annexin-V and propidium iodide after 24 hours of treatment with Ku0063794 or temsirolimus and then analyzed by flow cytometry. There was no evidence of apoptosis due to drug treatment (Fig. 4D). Apoptosis, indicated by positive Annexin-V and negative propidium iodide staining, was only seen in the positive control, which was treated with H_2_O_2_. We also evaluated Caspase 3, Caspase 9 and PARP1/2 in both Caki-1 and 786-O cells with drug treatment, and no protein cleavage was noted (data not shown); therefore, we saw no evidence of apoptosis.

### Ku0063794 Inhibits Tumor Growth and mTOR Signaling in a Xenograft Model of RCC

**Figure 5:**
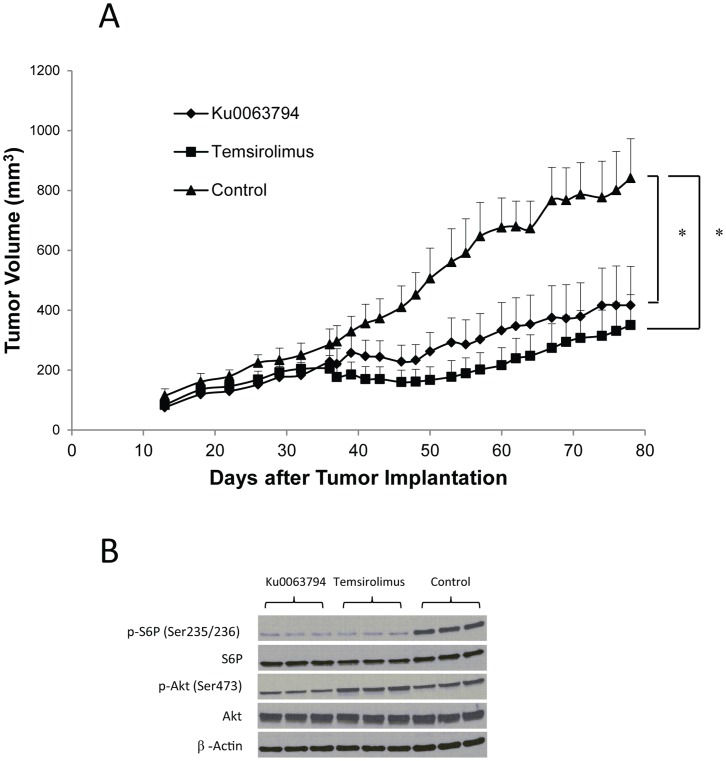
Ku0063794 and temsirolimus inhibited tumor growth in a xenograft model of RCC. (**A**) The treatment with Ku0063794 or temsirolimus significantly inhibited tumor growth in Nu/Nu nude mice. Following subcutaneous injection of 786-O cells, mice (5 mice in each group) were treated from days 33–78. The error bars indicate the standard error of the mean. *p<0.05 when the Ku0063794 or temsirolimus group was compared with control. (**B**) 90 minutes after drug administration, both Ku0063794 and temsirolimus inhibited the mTORC1 pathway *in vivo* as indicated by decrease in S6P phosphorylation in the tumor tissues, while only Ku0063794 inhibited the mTORC2 pathway *in vivo* as indicated by decrease in Akt phosphorylation on Ser473. Each lane represents a tumor from a different mouse.

Ku0063794 activity was investigated *in vivo*. To identify the maximum tolerated dose of Ku0063794, Nu/Nu nude mice were treated with a series of increasing daily doses of Ku0063794 to identify the highest dose that does not produce death or weight loss (data not shown). Tumors were generated in Nu/Nu nude mice by subcutaneously implanting approximately 5×10^6^ 786-O cells into the right flanks. Mice were treated with the highest tolerated dose of Ku0063794 (8 mg/Kg) for 46 days. Control mice were treated with temsirolimus (0.6 mg/Kg) or vehicle control. Treatment with both Ku0063794 and temsirolimus resulted in significant inhibition of tumor growth when compared with the control (P<0.05, Fig. 5A). To confirm that Ku0063794 and temsirolimus were inhibiting *in vivo* signaling, tumors were harvested and subjected to western blot analysis. Both Ku0063794 and temsirolimus inhibited the mTORC1 pathway *in vivo* as indicated by a decrease in S6P phosphorylation while only Ku0063794 inhibited the mTORC2 pathway as indicated by a significant decrease in Akt phosphorylation on Ser473 (Fig. 5B & Fig. S5).

### Temsirolimus but not Ku0063794 has Antiangiogenic Effects

**Figure 6:**
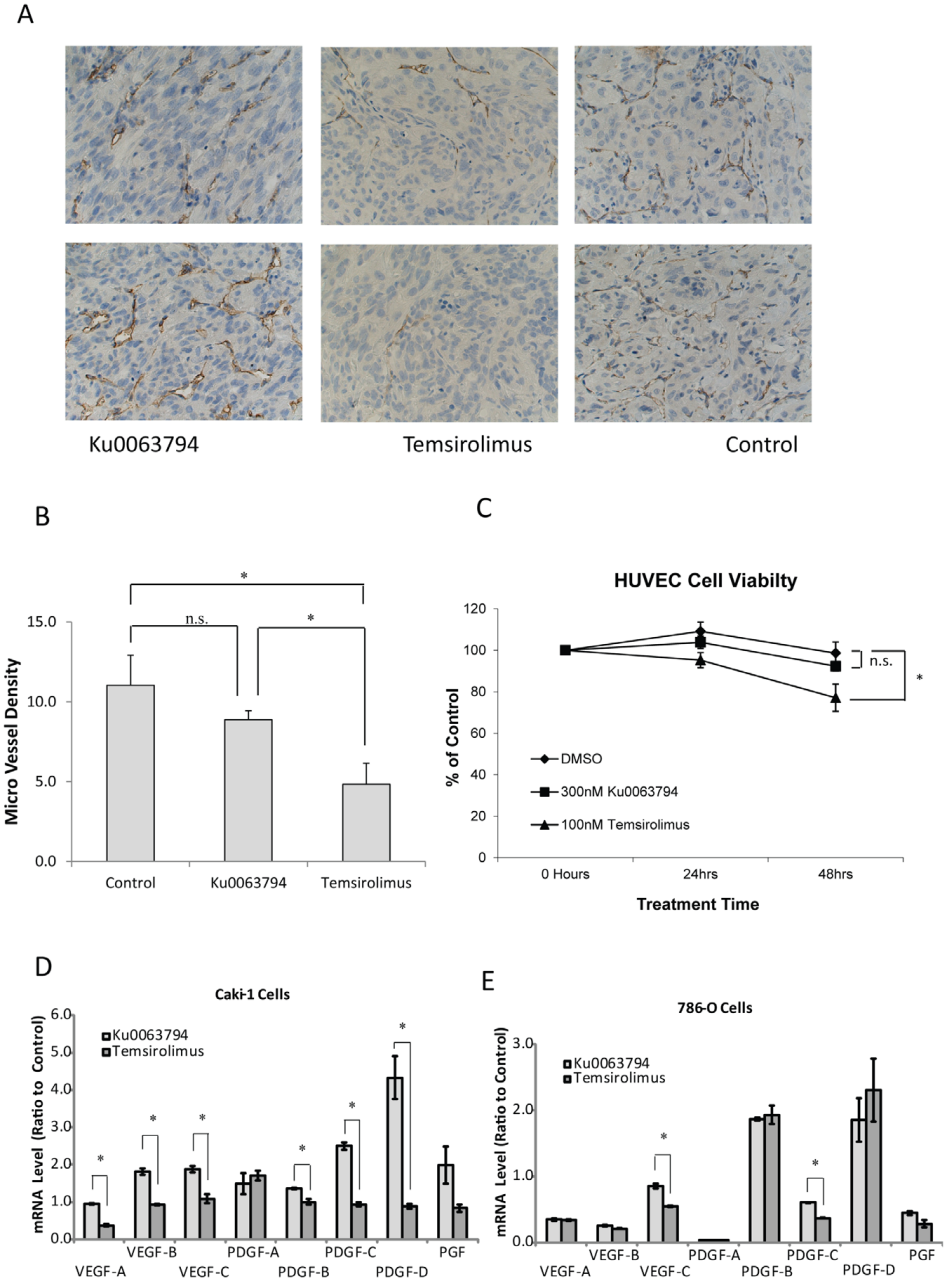
Temsirolimus but not Ku0063794 significantly inhibited tumor angiogenesis and viability of HUVEC cells at pharmacologically relevant concentrations. (**A**) Following subcutaneous injection of 786-O cells, mice were treated with temsirolimus, Ku0063794 or control. The resulting tumors were stained with CD34 antibody and 2 representative images for each treatment group are shown. (**B**) The micro vessel density (MVD) of the tumors was quantified from at least 4 mice per group. The error bars indicate the standard error of the mean. *p<0.05 when the temsirolimus group was compared with Ku0063794 or control. n.s., not significant. (**C**) *In vitro* cell viability assay was performed using HUVEC cells treated with Ku0063794 or temsirolimus for 24 to 48 hours at pharmacologically relevant concentrations. The error bars indicate the standard error of the mean for experiments performed in triplicate. *p<0.01 when the temsirolimus group was compared with the DMSO group (control). (**D, E**) The mRNA levels of various angiogenic growth factors were measured using TaqMan® PCR in Caki-1 and 786-O cells before and after 24 hours of treatment with Ku0063794 (2 µM) or temsirolimus (300 nM). Error bars indicate the standard error of the mean for experiments performed in triplicate. *p<0.05 comparing Ku0063794-induced and temsirolimus-induced expression changes.

Angiogenesis is an important target for treating advanced RCC. Therefore, we investigated the anti-angiogenesis effect of Ku0063794 and temsirolimus. Angiogenesis was evaluated in the xenograft tumors by CD34 immunohistochemical staining (Fig. 6A). Temsirolimus treatment significantly decreased tumor microvessel density (MVD) when compared to control tumors or tumors from mice treated with Ku0063794 (Fig. 6B). There was no significant difference in MVD when comparing the Ku0063794 treated group and the control group. To assess whether these drugs directly target endothelial cells, an *in vitro* cell viability study was performed using HUVEC cells, which are human endothelial cells. At pharmacologically relevant concentrations, temsirolimus decreased cell viability, but Ku0063794 did not (Fig. 6C). Pharmacologically relevant concentrations for temsirolimus were determined from clinical pharmacokinetic studies [24], [25]. Since we did not find any pharmacokinetic studies for Ku0063794, we selected a Ku0063794 concentration that produced similar effects on mTORC1 signaling as a pharmacologically relevant concentration of temsirolimus. An additional explanation for the difference in MVD is that temsirolimus treated tumors stimulate less angiogenesis (Fig. 6D & 6E). Consistent with this possibility, RCC cell lines treated with temsirolimus had lower expressions of angiogenic factors than RCC cell lines treated with Ku0063794. Caki-1 cells treated with temsirolimus had lower expression of VEGF-A/B/C and PDGF-B/C/D while 786-O cells had lower expression of VEGF-C and PDGF-C.

## Discussion

In all cancers, malignant transformation disrupts normal cellular metabolism. Genes linked to kidney cancer are involved in pathways that sense oxygen, energy and nutrient. The treatment of advanced RCC has been revolutionized by approval of small-molecule drugs that specifically target these biological pathways. mTOR is a central node in a cell’s metabolic pathway, receiving input from sensors of energy, nutrient and stress, and producing output that regulates protein synthesis and cell growth. mTOR inhibitors such as temsirolimus and everolimus are already FDA-approved for clinical use. These first generation mTOR inhibitors are rapamycin analogs that primarily target mTORC1. In phase III trials, both agents were shown to prolong progression free survival in patients with metastatic RCC and temsirolimus prolonged overall survival, validating the mTOR pathway as an important target for the treatment of RCC [8], [10].

In clear cell RCC there is a strong rationale for targeting both mTORC1 and mTORC2. VHL inactivation is found in the majority of clear cell RCC and results in constitutive activation of HIF regulated genes such as VEGF and PDGF [26], [27]. Both mTORC1 and mTORC2 have been shown to regulate the expression of HIF1α, however, mTORC2 appears to regulate HIF2α [28]. In normal cells, HIF1α is the critical isoform regulating the response to hypoxia. In clear cell RCC, HIF2α appears to drive tumor progression [29], [30]. Therefore, the inhibition of both mTORC1 and mTORC2 has the potential to be highly effective for inhibiting clear cell RCC. Consistent with this possibility, we found that clinical renal tumors had increased expression of genes associated with mTOR activity that were both sensitive and insensitive to mTORC1 inhibition. Cho et al reported that a second generation mTOR inhibitor targeting mTOR and PI3 Kinase decreased the level of HIF2α, while rapamycin did not [31].

Ku0063794 is a second generation mTOR inhibitor targeting mTORC1 and mTORC2. Ku0063794 was compared with temsirolimus using preclinical models of RCC. The 786-O cells are VHL^−/−^ and have constitutive HIF activity while Caki-1 cells are VHL^+/+^ [32]. These are two widely used human RCC lines that are documented to be derived from the clear cell variant of RCC. Table S1 summarizes the results of cell signaling studies. In human RCC cell lines, Ku0063794 inhibited the activity of both mTORC1 and mTORC2, while temsirolimus activity was generally limited to mTORC1. Our study suggests that phosphorylation of mTOR at Ser2448 and Ser2481 is primary regulated by mTORC2 since phosphorylation was strongly inhibition by Ku0063794 but not temsirolimus. However, prior reports do not firmly assign these phosphorylation sites to mTORC2 [4]. Our results also suggest that Ser2448 and Ser2481 of mTOR may not accurately reflect either mTORC1 or mTORC2 activity since phosphorylation of targets downstream of mTOR (e.g. Thr389 on p70 S6k, Ser473 on AKT, Ser21 on GSK-3α) preceded phosphorylation of Ser2448 and Ser2481. In our study, temsirolimus produced a transient decrease in the phosphorylation of AKT on Ser473 and Thr308, which are considered mTORC2 phosphorylation sites. This suggests that temsirolimus has some direct or indirect effect on this particular mTORC2-regulated phosphorylation. The effect may be brief because mTORC1 inhibition removes negative feedback loops targeting AKT; and increased AKT activity quickly overcomes any minor mTORC2 inhibition provided by temsirolimus.

*In vitro* cell viability studies were used to assess the direct effect of Ku0063794 and temsirolimus on human RCC cell lines. Ku0063794 decreased the viability of RCC cell lines in both a concentration and time dependent manner. In contrast, increasing the concentration of temsirolimus had a relatively small effect on cell viability, even though the concentrations tested included pharmacologically relevant concentrations. These observations suggest that Ku0063794 is a cytotoxic drug while temsirolimus is a cytostatic drug. This observation suggests that achieving the highest possible dose in phase one trials may be critical for second generation mTOR inhibitors.

Potential mechanisms resulting in decreased cell viability were examined. Both agents produced cell cycle arrest. Temsirolimus and Ku0063794 induced a marker of autophagy in the human RCC lines, and this agrees with a recent report by Chresta et al on a different dual mTOR inhibitor, AZD8055, which induces autophagy in human lung carcinoma cell lines [13]. Rapamycin is the canonical mTOR inhibitor and is well known to induce autophagy [33], [34]. However, it remains to be defined whether autophagy is directly leading to decreased cell viability or is a secondary response to another source of cellular stress directly induced by the drugs. Many cytotoxic agents induce apoptosis; however, neither Ku0063794 nor temsirolimus appears to induce apoptosis. Two recent reports examined two different dual mTOR inhibitors, AZD8055 and NVP-BEZ235 [13], [31]. No information was provided regarding the effect of AZD8055 on apoptosis. NVP-BEZ235 did not induce apoptosis in RCC cells *in vitro* but induced apoptosis in RCC xenograft tumors *in vivo* [29]. Our results suggest that Ku0063794 and temsirolimus decrease the viability of RCC cells by inducing cell cycle arrest and autophagy.

In our *in vivo* tumor-growth study, both temsirolimus and Ku0063794 significantly inhibited the growth of xenograft tumors. Ku0063794 appeared to have greater activity when directly applied to tumor cell lines *in vitro*. Therefore, it was surprising that Ku0063794 was not more effective than temsirolimus in the animal study. This is in contrast to a report by Cho et al, which showed that NVP-BEZ235 exhibited stronger inhibitory effect than rapamycin on the growth of RCC xenografts in a mouse model [31]. The difference may have resulted from subtle differences in dosing strategy, and differences in pharmacokinetics and metabolism of the drug analogs. However, it is important to note that in our study the maximum tolerated dose of Ku0063794 was used and inhibition of mTOR signaling was verified in the mouse tumors. Another important difference between Ku0063794 and NVP-BEZ235 is that NVP-BEZ235 is a much stronger inhibitor of PI3K than Ku0063794, and PI3K inhibition may be important for RCC [16], [35], [36].

A possible explanation for lack of greater activity *in vivo* for Ku0063794 is that temsirolimus has important effects on the tumor microenvironment. Temsirolimus decreased angiogenesis in the xenograft tumors while Ku0063794 did not. Further support for this possibility comes from our *in vitro* observation that temsirolimus decreased the viability of human endothelial cells while Ku0063794 did not. Temsirolimus treated tumors expressed less VEGF and PDGF than Ku0063794 treated tumors, thus stimulating less angiogenesis. In a separate study, our group has shown that temsirolimus can enhance antitumor immunity primarily by enhancing the formation of long-lived antitumor memory lymphocytes [37]. These studies show that first generation mTOR inhibitors may have important indirect effects that ultimately inhibit tumor growth. It is possible that second generation mTOR inhibitors lack the ability to favorably modulate host factors, which are an important consideration when evaluating new agents. Our results also provide a rationale for combining second generation mTOR inhibitors with antiangiogenic agents.

## Supporting Information

Figure S1 The western blots in Figure 2 for 1-hour treatment of both cell lines with both drugs were quantified with ImageJ software. *p<0.05 comparing treatment groups based on 2 representative blots.
(TIF)

Figure S2 Experimental replicates for western blots in Figure 2 for 1-hour treatment of both cell lines with both drugs.
(TIF)

Figure S3 The western blots in Figure 4 were quantified with ImageJ software. *p<0.05 comparing treatment groups based on 3 representative blots.
(TIF)

Figure S4 Experimental replicates for western blots in Figure 4.
(TIF)

Figure S5 The western blots in Figure 5B were quantified with ImageJ software. *p<0.05 comparing treatment groups based on 3 representative blots.
(TIF)

Table S1 Primer/Probe Sequences for Quantitative RT PCR.
(PPT)

Table S2 Summary of Intracellular Signaling Study.
(PPT)

Table S3 Summary of Cell Viability Assays.
(PPT)
