# Supplementary figures and images for: A Comparison of Ku0063794, a Dual mTORC1 and mTORC2 Inhibitor, and Temsirolimus in Preclinical Renal Cell Carcinoma Models
Source: PLoS One. 2013 Jan 22;8(1):e54918. doi: 10.1371/journal.pone.0054918 (PMC3551765; doi:10.1371/journal.pone.0054918)

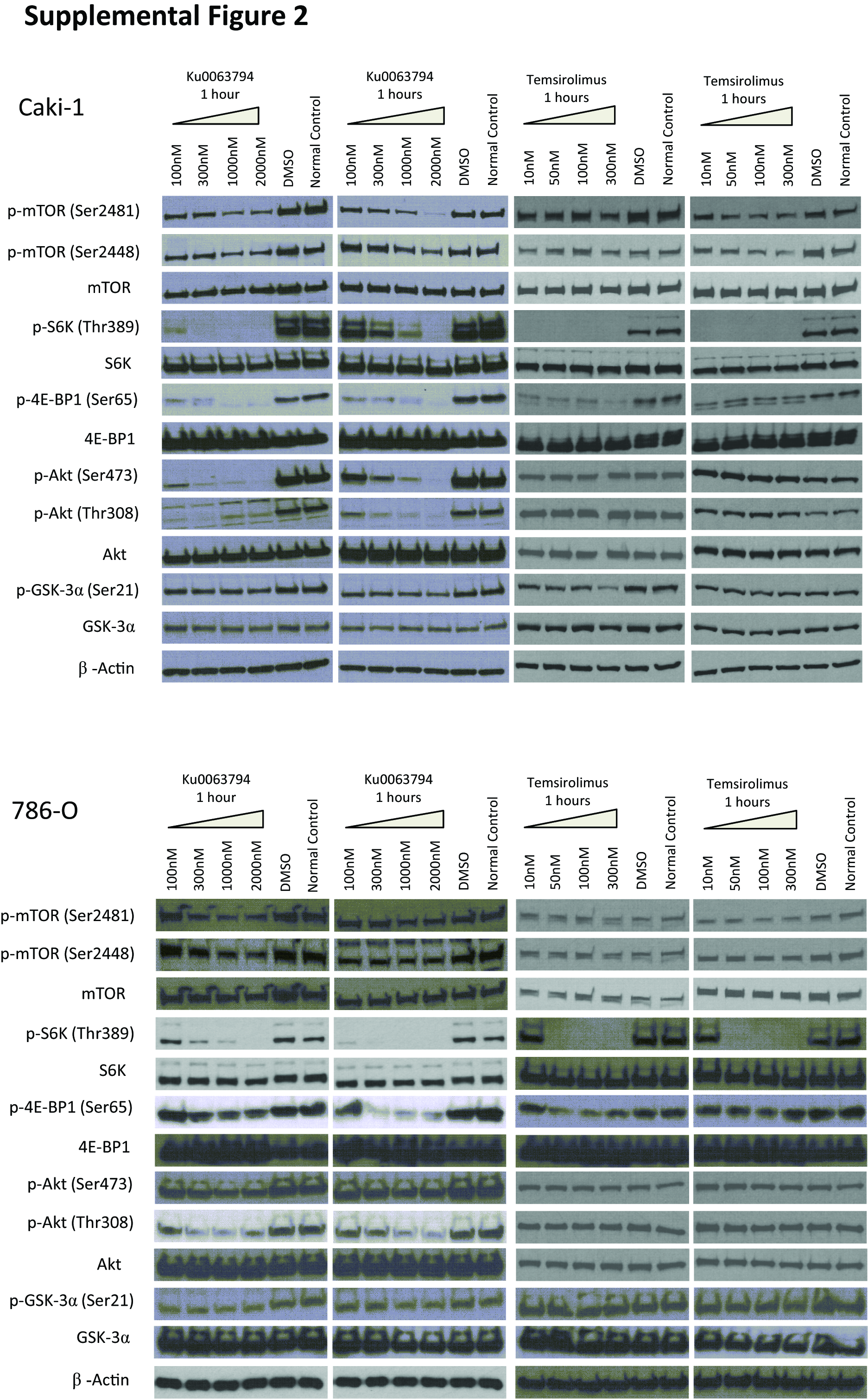

Supplement: Figure S2 — Experimental replicates for western blots in Figure 2 for 1-hour treatment of both cell lines with both drugs. (TIF) [file pone.0054918.s002.tif]

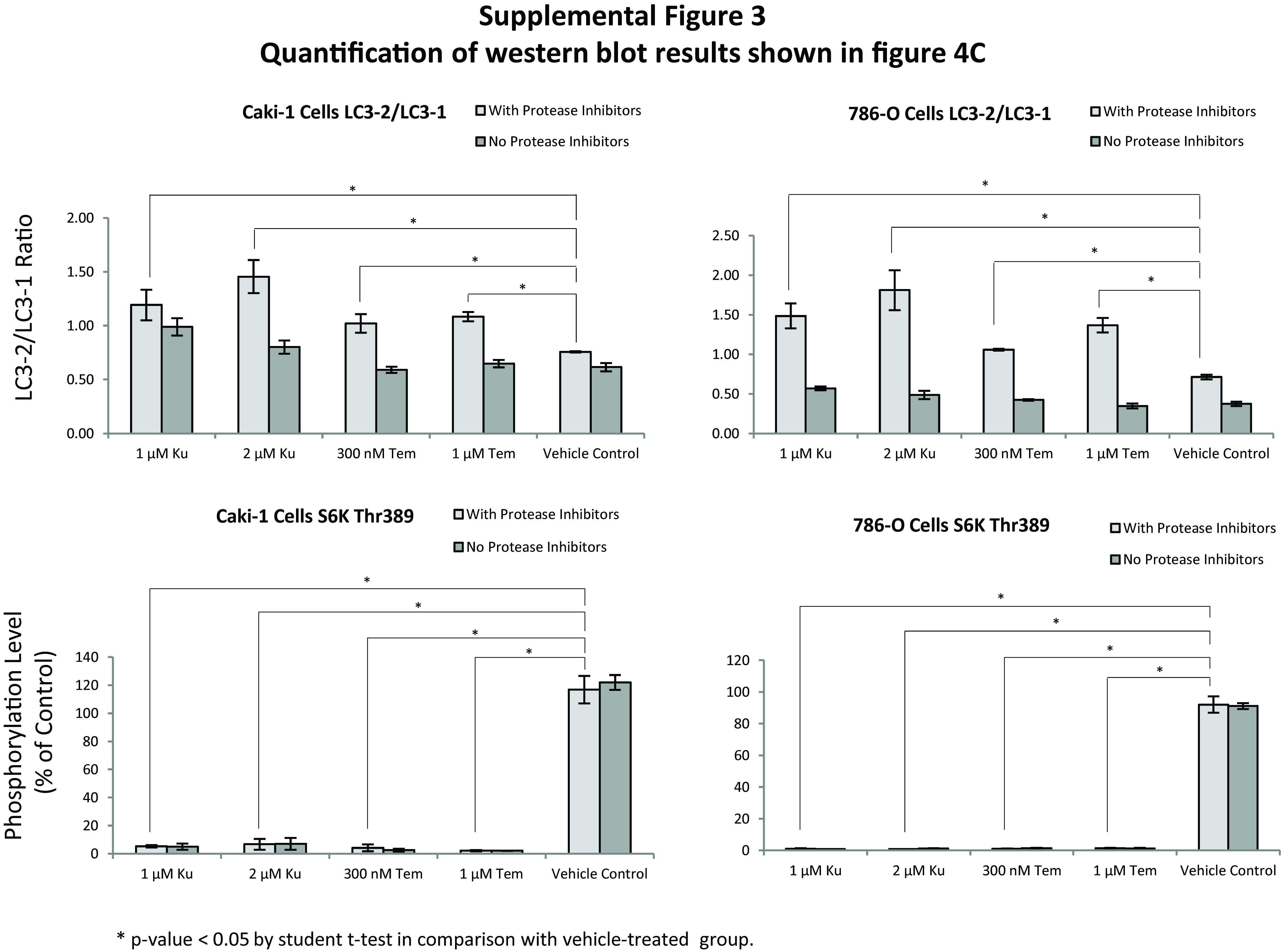

Supplement: Figure S3 — The western blots in Figure 4 were quantified with ImageJ software. *p<0.05 comparing treatment groups based on 3 representative blots. (TIF) [file pone.0054918.s003.tif]

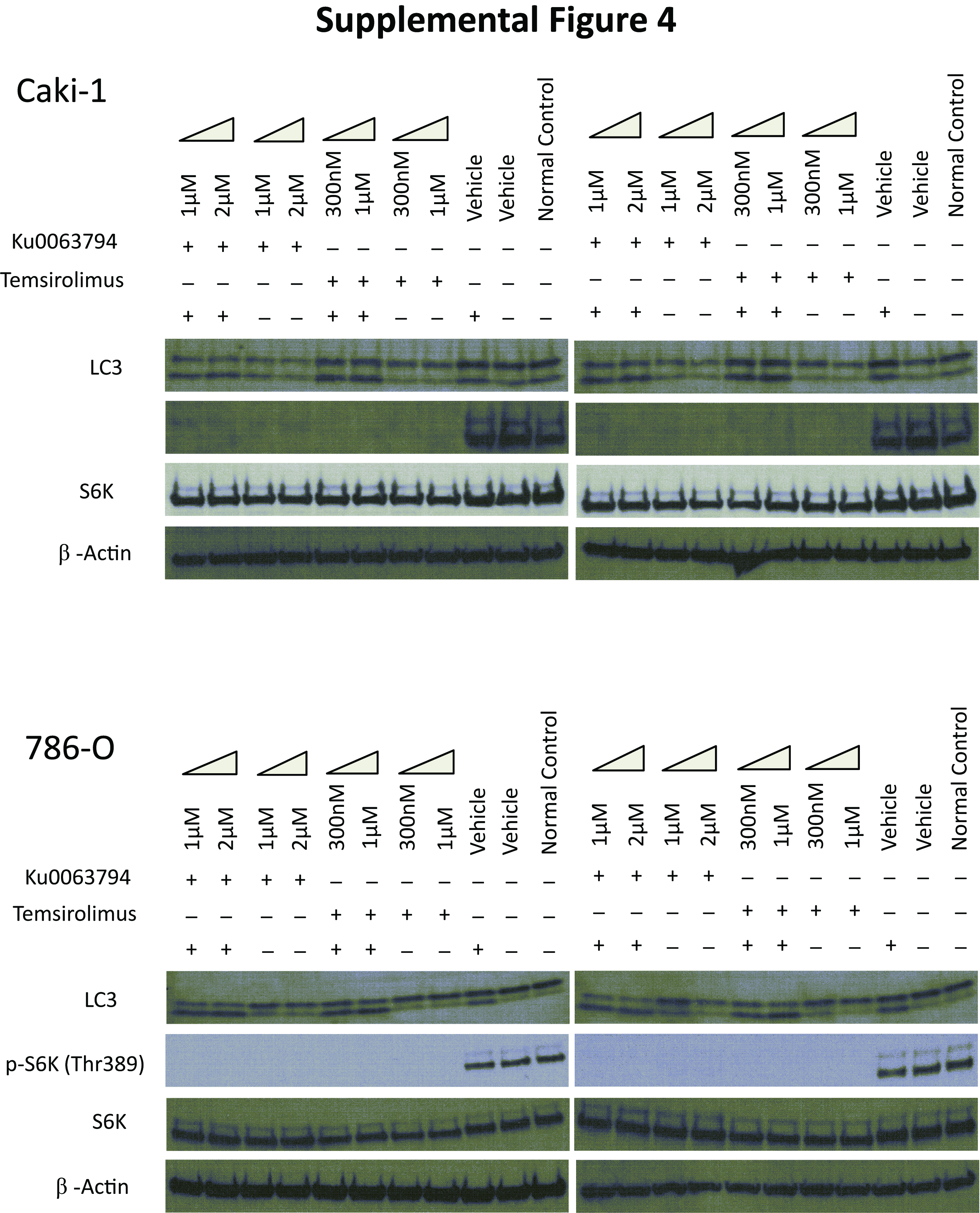

Supplement: Figure S4 — Experimental replicates for western blots in Figure 4. (TIF) [file pone.0054918.s004.tif]

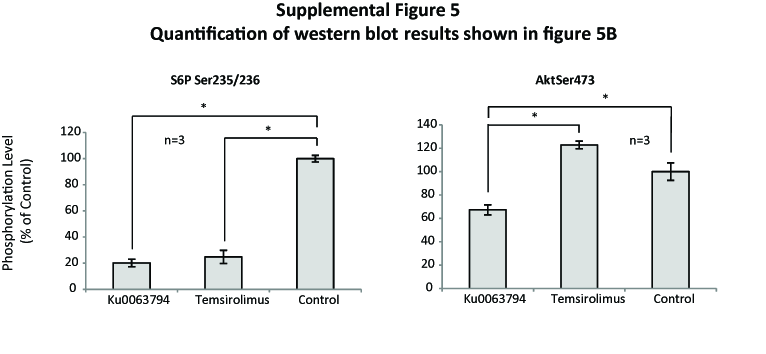

Supplement: Figure S5 — The western blots in Figure 5B were quantified with ImageJ software. *p<0.05 comparing treatment groups based on 3 representative blots. (TIF) [file pone.0054918.s005.tif]
