# Supplementary material for: A Comparison of Ku0063794, a Dual mTORC1 and mTORC2 Inhibitor, and Temsirolimus in Preclinical Renal Cell Carcinoma Models
Source: PLoS One. 2013 Jan 22;8(1):e54918. doi: 10.1371/journal.pone.0054918 (PMC3551765; doi:10.1371/journal.pone.0054918)
Supplement: Table S1 — Primer/Probe Sequences for Quantitative RT PCR. (PPT) [file pone.0054918.s006.ppt]

## Slide 1
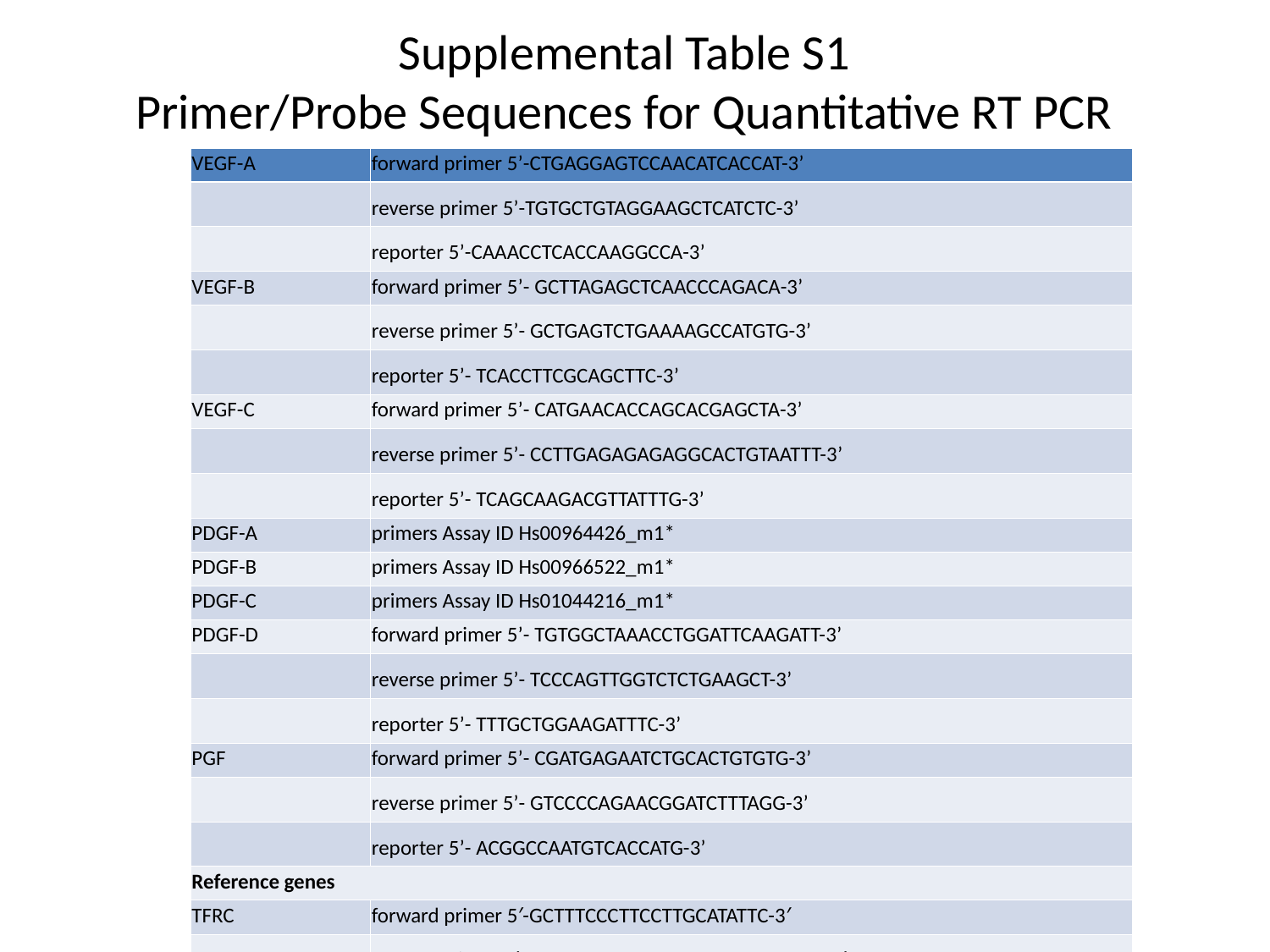

# Supplemental Table S1 Primer/Probe Sequences for Quantitative RT PCR
| VEGF-A | forward primer 5’-CTGAGGAGTCCAACATCACCAT-3’ |
| --- | --- |
| | reverse primer 5’-TGTGCTGTAGGAAGCTCATCTC-3’ |
| | reporter 5’-CAAACCTCACCAAGGCCA-3’ |
| VEGF-B | forward primer 5’- GCTTAGAGCTCAACCCAGACA-3’ |
| | reverse primer 5’- GCTGAGTCTGAAAAGCCATGTG-3’ |
| | reporter 5’- TCACCTTCGCAGCTTC-3’ |
| VEGF-C | forward primer 5’- CATGAACACCAGCACGAGCTA-3’ |
| | reverse primer 5’- CCTTGAGAGAGAGGCACTGTAATTT-3’ |
| | reporter 5’- TCAGCAAGACGTTATTTG-3’ |
| PDGF-A | primers Assay ID Hs00964426\_m1\* |
| PDGF-B | primers Assay ID Hs00966522\_m1\* |
| PDGF-C | primers Assay ID Hs01044216\_m1\* |
| PDGF-D | forward primer 5’- TGTGGCTAAACCTGGATTCAAGATT-3’ |
| | reverse primer 5’- TCCCAGTTGGTCTCTGAAGCT-3’ |
| | reporter 5’- TTTGCTGGAAGATTTC-3’ |
| PGF | forward primer 5’- CGATGAGAATCTGCACTGTGTG-3’ |
| | reverse primer 5’- GTCCCCAGAACGGATCTTTAGG-3’ |
| | reporter 5’- ACGGCCAATGTCACCATG-3’ |
| Reference genes | |
| TFRC | forward primer 5′-GCTTTCCCTTCCTTGCATATTC-3′ |
| | reverse primer 5′-GGTGGTACCCAAATAAGGATAATCTG-3′ |
| | reporter 5′-AATCCCAGCAGTTTCTTTCTGTTTTTGCGA-3 |
| B2M | forward primer 5′-TGAGTGCTGTCTCCATGTTTGA-3′ |
| | reverse primer 5′-CCACCTCTAAGTTGCCAGCC-3′ |
| | reporter 5′-TCCTAGAGCTACCTGTGGAGCAACCTGC-3′ |
| TBP | forward primer 5′-GCCCGAAACGCCGAATA-3′ |
| | reverse primer 5′-CGTGGCTCTCTTATCCTCATGA-3′ |
| | reporter 5′-CCCAAGCGGTTTGCTGCGGT-3′ |
