# Supplementary material for: A Comparison of Ku0063794, a Dual mTORC1 and mTORC2 Inhibitor, and Temsirolimus in Preclinical Renal Cell Carcinoma Models
Source: PLoS One. 2013 Jan 22;8(1):e54918. doi: 10.1371/journal.pone.0054918 (PMC3551765; doi:10.1371/journal.pone.0054918)
Supplement: Table S2 — Summary of Intracellular Signaling Study. (PPT) [file pone.0054918.s007.ppt]

## Slide 1
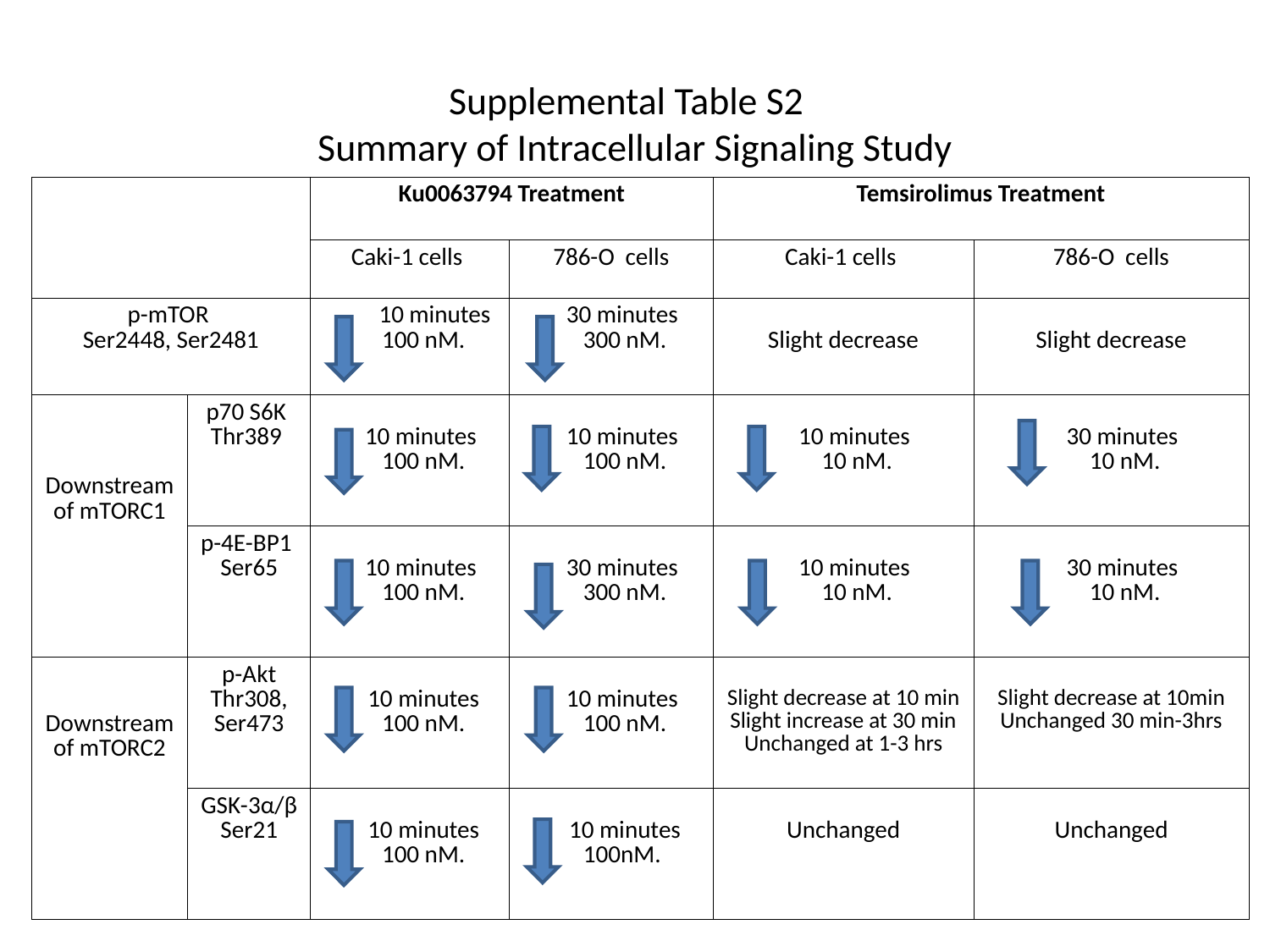

# Supplemental Table S2 Summary of Intracellular Signaling Study
| | | Ku0063794 Treatment | | Temsirolimus Treatment | |
| --- | --- | --- | --- | --- | --- |
| | | Caki-1 cells | 786-O cells | Caki-1 cells | 786-O cells |
| p-mTOR Ser2448, Ser2481 | | 10 minutes 100 nM. | 30 minutes 300 nM. | Slight decrease | Slight decrease |
| Downstream of mTORC1 | p70 S6K Thr389 | 10 minutes 100 nM. | 10 minutes 100 nM. | 10 minutes 10 nM. | 30 minutes 10 nM. |
| | p-4E-BP1 Ser65 | 10 minutes 100 nM. | 30 minutes 300 nM. | 10 minutes 10 nM. | 30 minutes 10 nM. |
| Downstream of mTORC2 | p-Akt Thr308, Ser473 | 10 minutes 100 nM. | 10 minutes 100 nM. | Slight decrease at 10 min Slight increase at 30 min Unchanged at 1-3 hrs | Slight decrease at 10min Unchanged 30 min-3hrs |
| | GSK-3α/β Ser21 | 10 minutes 100 nM. | 10 minutes 100nM. | Unchanged | Unchanged |
