# Supplementary material for: A Comparison of Ku0063794, a Dual mTORC1 and mTORC2 Inhibitor, and Temsirolimus in Preclinical Renal Cell Carcinoma Models
Source: PLoS One. 2013 Jan 22;8(1):e54918. doi: 10.1371/journal.pone.0054918 (PMC3551765; doi:10.1371/journal.pone.0054918)
Supplement: Table S3 — Summary of Cell Viability Assays. (PPT) [file pone.0054918.s008.ppt]

## Slide 1
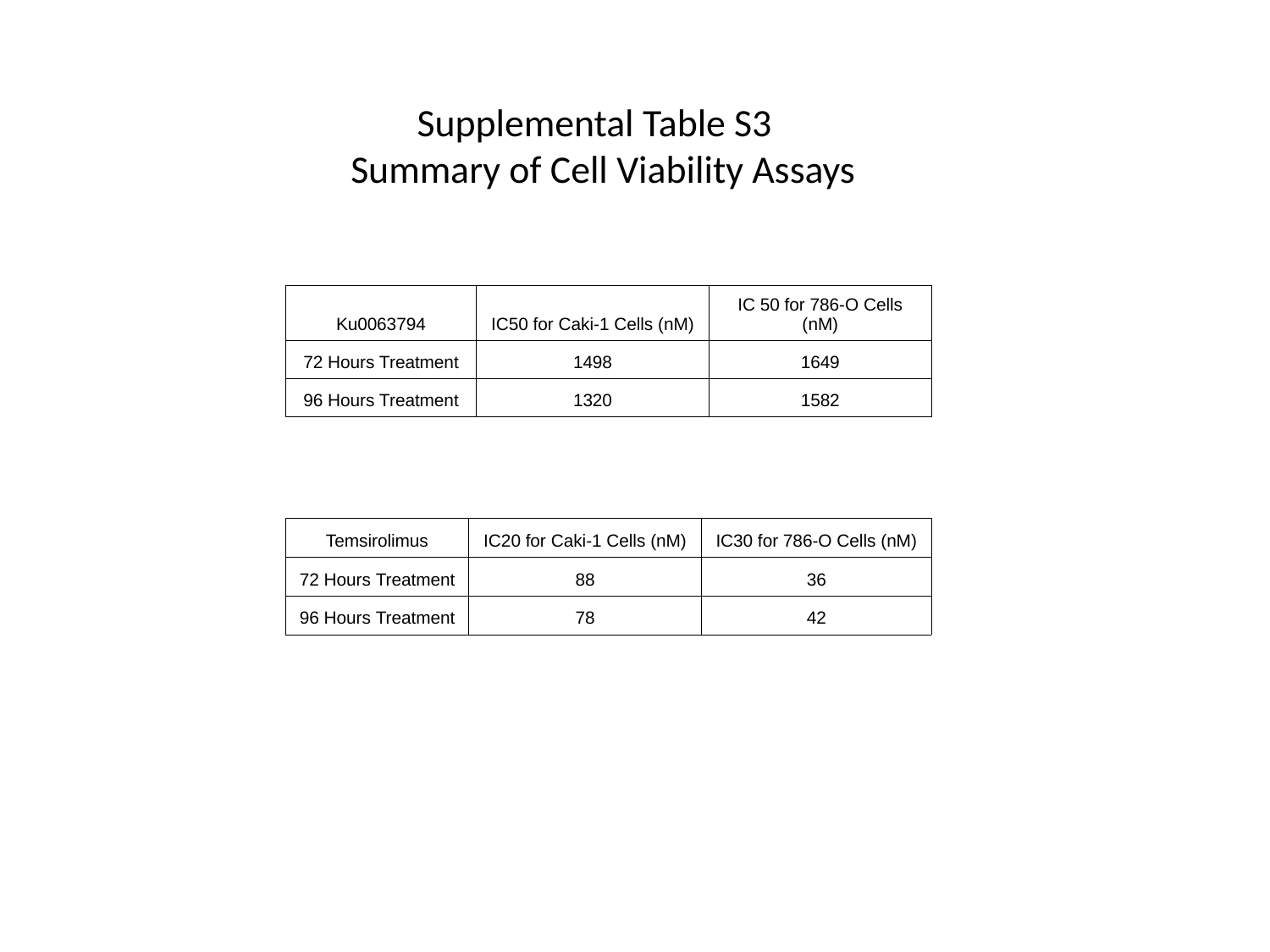

Supplemental Table S3
Summary of Cell Viability Assays
| Ku0063794 | IC50 for Caki-1 Cells (nM) | IC 50 for 786-O Cells (nM) |
| --- | --- | --- |
| 72 Hours Treatment | 1498 | 1649 |
| 96 Hours Treatment | 1320 | 1582 |
| Temsirolimus | IC20 for Caki-1 Cells (nM) | IC30 for 786-O Cells (nM) |
| --- | --- | --- |
| 72 Hours Treatment | 88 | 36 |
| 96 Hours Treatment | 78 | 42 |
